# Epidemiology of adult asthma within the Hungarian population between 2009 and 2019 – A retrospective financial database analysis

**DOI:** 10.1016/j.gloepi.2025.100191

**Published:** 2025-03-06

**Authors:** B. Sánta, A. Keglevich, T. Kovács, B. Engi, A. Südi, E. Noémi, L. Tamási

**Affiliations:** a Chiesi Hungary Kft, Hungary; b Semmelweis University, Department of Pulmonology, Hungary

**Keywords:** Asthma epidemiology, Asthma treatment, Healthcare resource allocation

## Abstract

**Background:**

Asthma is the most common chronic obstructive respiratory disease and is a considerable burden on the patients, caregivers and healthcare providers. However, data on epidemiology, healthcare expenditures, inhalation medication usage and comorbidities are scarce on a country-wide level.

**Methods:**

A retrospective analysis was performed on the Hungarian National Health Insurance Fund's financial database. All patients who had filled at least one prescription for asthma maintenance therapy between 2009-’19 had been enrolled. Prevalence for each year was assessed, based on prescriptions filled for asthma annually. Incidence was assessed only for 2011–’19. Frequency of exacerbations and their costs were calculated.

**Results:**

Through the study period 439,977 patients filled at least one prescription for asthma maintenance therapy. The number of patients having at least one prescription in 12 months increased by 20.34 % (from 132,292 to 159,225 patients). Between 2011–’19 an average of 20,742.1 new patients used asthma maintenance medications. Between 2009 and ‘19 an average of 4308 patients were hospitalized due to asthma, on an average 5129 times. Healthcare expenditure on hospitalizations and outpatient treatment of asthmatics increased by 28.05 %.

**Conclusion:**

Through our study period a substantial increase in patient numbers and overall expenditure was seen. Number and frequency of exacerbations however decreased over the years.

## Introduction

Asthma is the most prevalent non-communicable airway disease, which affected 262 million patients worldwide in 2019, according to the World Health Organization (WHO) with an even higher estimate in recent years according to the Global Initiative of Asthma (GINA) guidelines. [1,2] The prevalence of asthma is high both in childhood and adulthood as well, however large geographical differences can be observed. [3] In Europe, the reported asthma prevalence is 5.86 %, however there is a more than 7 % difference between the highest and lowest reported values on a country level. [3] According to estimations the prevalence of asthma had decreased considerably in the past 30 years in Europe, despite the increase of the prevalent cases of asthma. [4] Similarly, the incidence of asthma also decreased considerably, with a parallel increase in incident cases. [5] However, it is also important to note that reporting of asthma had changed over time multiple times. As multiple definitions had been in circulation and multiple ways of reporting asthma are followed in different countries, providing an accurate estimate proves challenging. [6]

Besides available global and regional reports, many countries worldwide and in Europe as well, initiated programs to follow the changes in demographics of asthma. Most of these programs aimed also at improving asthma related health outcomes, recognizing the huge societal effect of the disease. [7] Some of these registries from the largest European countries besides reporting on prevalence, also showed that far from all patients receive prescription treatment (average of 74.1 %, ranging from 67.2 % to 83.7 %). [8] Khan *et al* also reported on patients' perspective on their disease severity, showing a high perceived burden of disease. [8] This finding is very alarming considering the substantial development in asthma treatment in the past few decades. [9]

Considering a high prevalence of asthma, even with a lower per patient cost compared to other diseases [10,11], the overall economic burden of asthma is substantial. [12] These costs partly derive from the costs of treatment of asthma, like medications and hospital care (*direct* costs) and due to the loss of productivity of the patients and their caregivers and earlier mortality (*indirect* costs). The distribution of the overall costs between these types of expenditures can vary depending on the severity of asthma and on geography. [13,14] Generally, we can conclude that even though the proportion of patients having mild disease is much higher, the overall costs for severe patients can outweigh that of patients' with milder disease. [13]

Among the direct costs, the main drivers of expenditures are hospitalizations for asthma and medication use. [15,16] As the GINA guidelines state, proper maintenance treatment can decrease the need for hospitalizations due to asthma. [2] It is safe to assume that with a higher use of medication the lower is the expected expenditure for hospitalizations and *vice versa*. Considering that, the avoidance of the need for hospitalizations due to asthma is one of the main goals of asthma management, [17] we can assume that in regions with high level of asthma care, the expenditures for hospitalizations also decrease, however there is a lack of factual knowledge in this topic.

Even with the improvements of reporting on asthma epidemiology and outcomes increase worldwide, there is a lack of in-depth knowledge on these parameters in Central-Eastern Europe. Also, as mentioned above, the reliability of certain databases is questionable, due to the high variability in definitions of asthma, there is a need for reliable, universal databases to help with resource allocation to improve asthma care. Hungary has a single insurer healthcare system, where almost the whole country's population is insured by the same entity, the National Health Insurance Fund (NHIF). The financial database of the NHIF is available for research on a claims basis, and had been used by our research group in earlier publications. [18,19]

Our aim was to investigate the demographic parameters of asthma patients under regular maintenance treatment, based in the financial database of the NHIF.

## Methods

### Data source

A retrospective financial database analysis was performed, using the database of the NHIF to select all adult asthma patients treated between 2009 and ‘19. As Hungary has a single insurer healthcare system, this database encompasses the whole population of Hungary, collecting data on all procedures and treatments, including medication prescriptions. However, data on the outcome of the procedures and medical information is not recorded. All prescription and outpatient procedures are coded, using the International Classification of Diseases 10th edition (ICD-10). Furthermore, in case of hospital admissions, an ICD-10 code is also added as the main diagnosis, describing the cause for admission. Due to privacy policy, no personal data of a single patient is available from the database. Further details of this database had been reported in our earlier studies. [18,19] Local study authorization was provided by the National Institution of Pharmacy and Nutrition of Hungary, based on the beneficial assessment by the National Scientific and Research Ethics Committee of Hungary (docket number: IV/8716–3 /2021/EKU).

### Cohort formation and statistics

Data of all patients who had at least one maintenance treatment prescription for the ICD-10 code of asthma (J45) had been collected between 01.01.2009 and 12.31.2019. The end of 2019 was chosen as the end of the study as our aim was to describe general characteristics of asthma patients from the past decade, and after 2020, the COVID-19 pandemic had a large disruptive effect on usual healthcare.

Patients were considered on-treatment asthmatics, if they had at least one maintenance treatment prescription for the ICD-10 of asthma in the overall study duration. In each year, on-treatment patients were the ones with at least one prescription for asthma maintenance therapy, with J45 code. Furthermore the following rule was applied: among the last 10 prescriptions of maintenance treatment, there could be no prescriptions with the ICD-10 code of COPD (J44), only asthma (J45). If a patient had less than 10 prescriptions during the study period (2009-’19) they could only be included if within one year the ratio of asthma per asthma plus COPD prescriptions of maintenance inhaler treatments was above 80 %. A strict, absolute exclusion of the J44 code would limit our population too much, considering that it would exclude patients with misclassifications and those with difficult differential diagnosis. Misclassification here means the faulty use of ICD-10 codes due to administrative error. This step was included as asthma and COPD may have similar characteristics that may lead to difficulty of differential diagnosis, sometimes, resulting in difficulty of differential diagnosis, oftentimes resulting in prescriptions with ICD-10 codes of both diseases, for a short period of time, until final diagnosis is made.

In Hungary, the network of outpatient clinics and hospital ambulatory care centers, and inpatient centers report on the numbers of diagnosed asthma patients every year. The summary of these reports are published in the publicly available Korányi Bulletin. [20] We used these numbers as reported asthma prevalence and incidence to compare to the on-treatment numbers of asthmatics.

Incident cases were identified based on prescription and treatment history: if a patient had no prescription for any medication nor had in-, or outpatient care with the ICD-20 code of asthma, before a given year, they were considered incident cases for that year. As we had no data before 2009, incidence was not evaluated for 2009 and 2010, taking into consideration that treatment cessation due to low adherence can be quite common. As there are many patients with mild asthma who might not need a prescription every year, it was decided that the first two years of the study, incidence will not be evaluated to increase accuracy.

Severe exacerbations were defined as a need for hospitalization due to asthma. Hospitalizations were considered related to asthma if the ICD-10 code of asthma was recorded as the main cause for inpatient care in the hospital records. Costs of care were calculated from the diagnosis-related group (DRG) value of COPD care.

To compare prevalence of asthma in each year, the publicly available database of the Hungarian Central Statistics Office was used, in order to calculate the number of adult populations in Hungary. [21]

The costs of hospitalizations for asthma are shown in EUR exchanges on the average exchange rate of EUR to HUF in 2009, according to the database of the European Central Bank. [22]

Only descriptive statistics were used to present data.

## Results

### Prevalence and incidence

Using the database of the NHIF, all adult patients who had filled a prescription for maintenance inhaled treatment with ICD-10 code of asthma, between January 1st, 2009 and December 31st, 2019 had been selected. Altogether 439,977 of such, on-treatment asthma patients were identified. The number of patients on-treatment with asthma increased from 132,292 in 2009 to 159,225 in 2019, meaning an overall 20.4 % increase. Crude average year-to-year increase of 1.90 % (95 %CI: 0.32 % to 3.48 %) was observed, compared to the previous year. During the same period, number of reported asthma patients, according to Korányi Bulletin increased from 248,165 patients in 2009 to 321,039 patients in 2019, overall 29.4 % % with an annual increase of 2.64 % (95 %CI: 0.84 % to 4.45 %), compared to the previous year. [20]

**Fig. 1:**
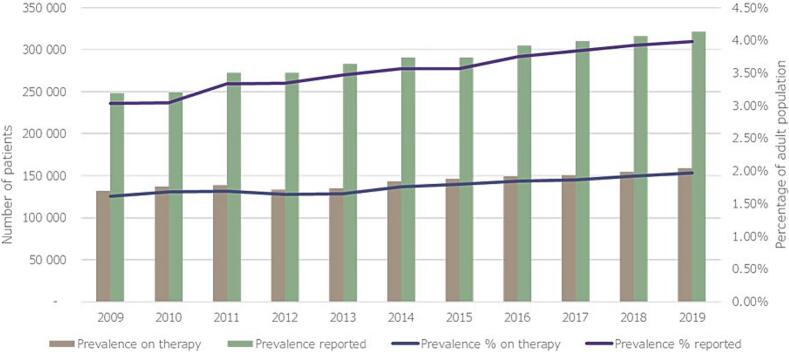
Annual number of reported and on therapy asthma patients in Hungary, and their respective prevalences in the adult population between 2009 and 2019.

Prevalence of treated asthma in the adult population increased from 1.62 % to 1.98 %, an overall 22.1 % increase, compared to the previous year. The year-to-year average increase was 2.04 % (95 %CI: 0.52 % to 3.56 %). During the same period of time prevalence of reported asthma increased from 3.03 % in 2009 to 3.58 % in 2019. Detailed increases in asthma patient numbers in Hungary and prevalences are shown in Fig. 1**.**

Number of newly diagnosed cases on treatment, decreased from 29,814 patients in 2011 to 17,112 patients in 2019, overall decrease of 42 %. Year-to-year average decreasedof the number of newly diagnosed patients was 6.14 % (95 %CI: −12.69 % to 0.42 %). At the same time, number of newly registered asthmatics decreased from 17,116 in 2011 to 13,675 in 2019, overall decrease of 20.1 %.

**Fig. 2:**
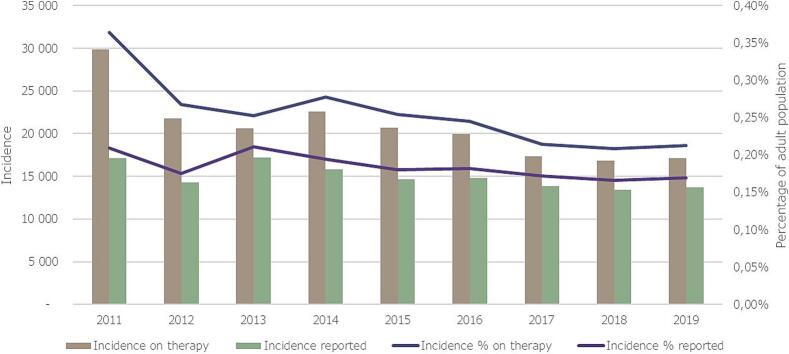
Annual number of newly reported and on therapy asthma patients in Hungary, and their respective incidences in the adult population between 2009 and 2019.

The corresponding incidence rates in the adult population showed a decrease from 0.35 % in 2011 to 0.21 % in 2019 for patients on therapy, while it decreased from 0.21 % to 0.18 % for reported new asthmatics. These values correspond to a yearly average annual decrease of −5.96 % (95 %CI: −12.47 % to 0.54 %) for treated asthmatics. Detailed changes in newly diagnosed patient numbers and incidences in the adult population are shown in Fig. 2**.**

### Distribution according to age and sex

Age distribution of asthma patients on therapy changed significantly, with a much smaller difference in gender distribution over the period of our study. In 2009 65.1 % of all patients were female, while in 2019 it was 66.1 %, meaning on overall 1.51 % increase, compared to baseline.

**Fig. 3:**
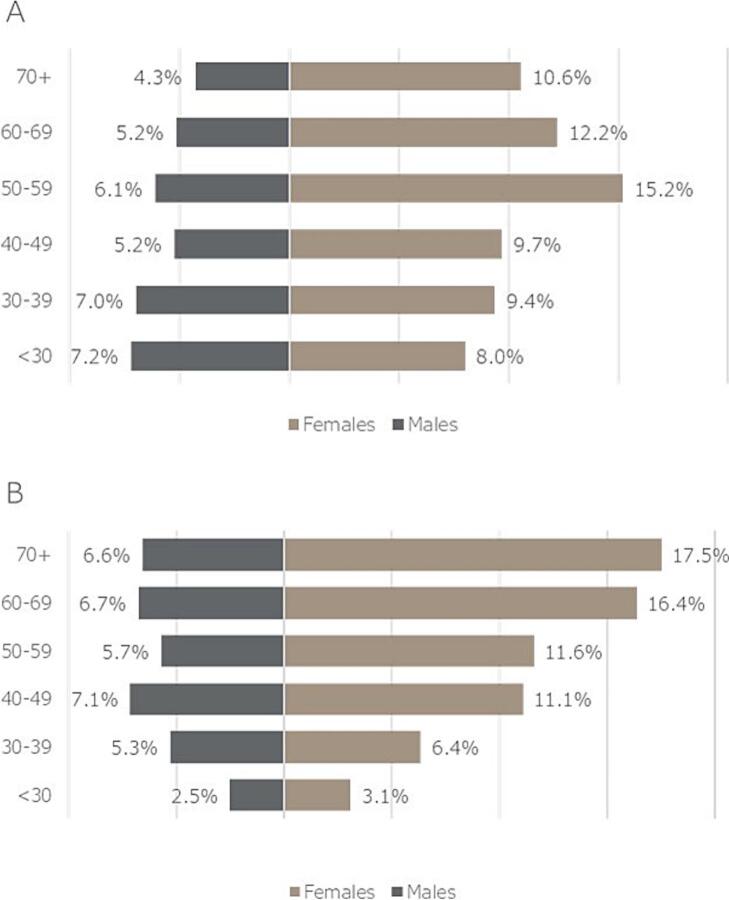
(a and b) Population pyramids of treated asthmatic patients in Hungary in 2009 (A) and 2019 (B).

Regarding age, the most prevalent age group was 50–59 years among females (15.2 %) in 2009, while the most prevalent group was 70 ≤ years in 2019 (24.1 %). Important changes were observed among males as well, with the most prevalent group being <30 years (7.2 %) in 2009 and patients 40–49 years (7.1 %) in 2019. The population pyramids of 2009 and 2019 are shown in Fig. 3a **and** 3**B.**

### Hospitalizations

**Fig. 4:**
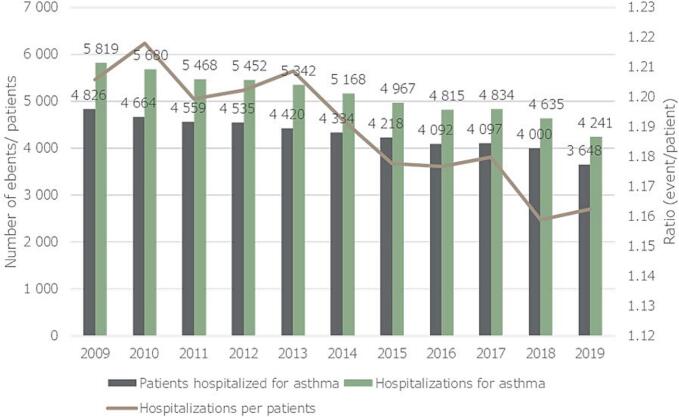
Number of asthma patients hospitalized for asthma, and number of hospitalizations, and their ratio between 2009-’19 in Hungary.

During our study, the number of hospitalizations for ICD-10 code of asthma (severe exacerbations) had decreased markedly, along with the number of patients hospitalized for asthma. In 2009, 4826 patients were hospitalized for asthma, altogether 5819 times, meaning that one asthma patient who is hospitalized for an exacerbation, on average, gets hospitalized 1.21 times. In 2019 however, only 3648 patients were hospitalized, altogether 4241 times (ratio of 1.16). This change translates to a crude average − 2.73 % (95 %CI: −4.21 to −1.25) decrease in terms of patients hospitalized for asthma, and a − 3.09 % (95 %CI: −4.59 to −1.58) decrease in terms of asthma hospitalizations year-to-year, compared to the previous year. Annual changes in hospitalizations are shown in Fig. 4**.**

Compared to the overall asthma population, an even larger decrease was observed. In 2009, 36.5 patients/1000 asthmatics were hospitalized for asthma, and 44.0 hospitalizations happened per 1000 asthma patients. In 2019 however only 22.9 asthmatics were hospitalized, and 26.6 hospitalizations happened due to a severe exacerbation per 1000 asthma patients. Crude annual decreased of the number of hospitalized asthma patients was −4.47 % (95 %CI: −6.93 to −2.01), while the number of hospitalizations for asthma decreased on average by −4.81 % (95 %CI: −7.37 to −2.25) every year, as compared to the previous year.

### Costs of hospitalizations

**Fig. 5:**
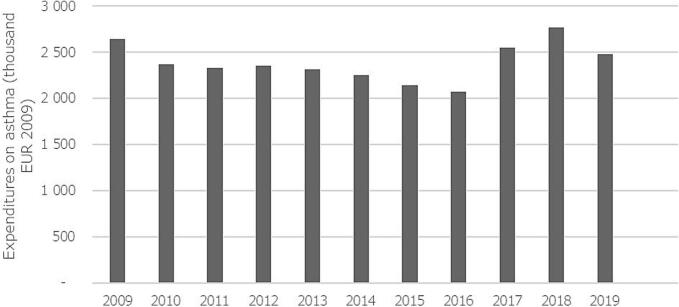
Costs of hospitalizations of asthmatics for asthma between 2009 and ‘19 in Hungary (all expenses are in EUR based on the exchange ratio of 2009).

The costs for hospitalizations due to asthma showed a marked decrease from 2009 until 2016, when a sharp increase was observed for 2 years and then the costs started decreasing again. Interestingly, during the same period of time, the costs of hospitalizations of asthma patients for any other diseases increased steadily, with a steep increase between 2016 and 2018. During the study period, the overall costs for asthma exacerbations decreased by −6.39 %, with a crude average annual decrease of −0.25 % (95 %CI: −6.36 to 5.86) compared to the previous year. At the same time, the costs of any other hospitalizations increased by 72 %, with an crude annual average increase of 5.73 % (95 %CI: 2.00 % to 9.47 %) compared to the previous year. Details of the annual costs are shown in Fig. 5**.**

## Discussion

Our study showed that during the last decade, there was a large increase in the overall number of asthma patients who use inhaled maintenance treatment regularly. At the same period, the number of newly diagnosed asthmatics decreased markedly, even compared to the decreasing population of Hungary. During this time the number of hospitalized asthmatics and the number of hospitalizations also decreased markedly, especially compared to the growing numbers of asthmatics. Our study also revealed that the overall costs of asthma hospitalizations showed a decrease along with the number of events, however the costs of hospitalizations for any other reason increased markedly during the same period.

An important addition of our study to our current knowledge is the distinction of diagnosed (and reported) asthmatics and asthma patients who actually use their medication on a regular basis. As compared to the numbers reported in Korányi Bulletin [20], our study showed that on average about 50 % of all diagnosed asthma patients use maintenance inhalation treatment on a regular basis. This definition excludes patients with mild disease who might not need to use more than one prescription of maintenance inhalation treatment annually, and patients with minimal adherence to treatment, who despite being diagnosed might not visit their doctors or not fill their prescriptions.

It is also important to highlight that even considering the prevalence of diagnosed asthmatics, the prevalence is below 4 % of the adult population, which is far from the accepted estimate. [3] However, considering the number of patients actually treated regularly with maintenance asthma treatments, this percentage is even lower, highlighting the need for a closer focus on asthma from the medical society in Hungary. Asthma is generally considered an underdiagnosed disease globally, and our study further highlights this fact.

The number of newly diagnosed asthmatics in Hungary is even though high, showed a relevant decrease over the study period, even when compared to the shrinking overall population of the country. Although the disease is underdiagnosed, it seems to be harder and harder to diagnose a new asthma patient. This is most likely due the underperformance of the healthcare system stemming from a lack of resources and increasing burden of patients' treatment. The OECD annual report showed that the number of active healthcare providers did not grow in the past years, [23] yet the number of patients with pulmonary problems, such as COPD, asthma and lung cancer increased at a steady rate. [20] Our study aimed at assessing the demographics of asthma before the COVID pandemic, but looking at the results of the Korányi Bulletin between 2020 and 2022, it is quite clear that the addition of further patients (*e.g.*: diagnosis and treatment of COVID) has a very detrimental effect on the recognition of populational diseases. [20] Assuming that this trend continues, it is likely that the number of newly diagnosed asthmatics will plateau.

Similarly to global trends, the number of hospitalizations due to asthma decreased in the past decade, which is most likely the result of improvements in treatment. This improvement in one hand stems from the universal introduction of inhaled corticosteroids (ICS) to maintenance treatment, and from the more and more prevalent routine use of treatment guidelines, which help healthcare providers find the best solution for populational diseases. However, it is important to highlight that even in 2019 more than 3600 patients were hospitalized for asthma (more than 10 daily) and that one asthma patients who had been hospitalized is most likely will be hospitalized again within the same year. In one of our previous studies, using the same database, we showed that hospitalizations are strong predictive factors for further need for hospital care in asthmatic patients. [19] These numbers show that even though asthma care is improving the treatment of asthma is still not a resolved issue.

Finally, our study highlights the high burden of comorbidities on asthmatics considering that annually, on average the healthcare system spends 20 times more on hospital care for other diseases than asthma for asthmatics. In our previous study a composite comorbidity score, the Charlson index was one of the most important predictors of death after hospitalizations. [19] Also in another local study, it was shown that many comorbidities increase the chance of uncontrolled asthma, thus most likely increasing the need for hospitalizations as well. [24]

The strengths of our study are the high number of enrolled patients, the high reliability and accuracy of the NHIF's database and the almost 100 % coverage of the Hungarian population in the database. The limitations are the lack of clinical results, like spirometry, symptoms scores, and details of hospitalizations to assess severity of asthma. Furthermore the use of simple, descriptive statistics is another limitation of our results. Also, as the definitions used had not been validated, we cannot ensure that all enrolled patients were in fact asthmatics and that we did not exclude asthmatic patients in our attempt to exclude COPD patients. However, we choose prescription data as our diagnostic criteria, as it is our belief that if a doctor prescribes a medication with a certain diagnostic code, it is more likely to be accurate, compared to simple outpatient visits. Also, data collection was stopped at the end of 2019 for the goal of excluding the effects of the COVID-19 pandemic on healthcare. However, the disruption of routine care and the following rebuilding period could also show important details not just on the care of asthmatics but the adaptability of the overall healthcare system as well.

## Conclusions

Our study showed that asthma has a growing prevalence in Hungary, despite the shrinking of the country's overall population, and that despite improvements of asthma care, it is still an unresolved problem, causing over 3600 hospitalizations annually. Our study also proved that asthmatics have a high comorbidity burden, so the management of comorbid diseases is paramount for optimal asthma care. At the same time, the decreasing number of hospitalizations indicates an improvement in asthma care in Hungary.

## Contribution

All authors made a significant contribution to the work reported. BS in the conception, formal analysis, investigation, acquisition of data, took part in the drafting of the manuscript; AK, TK, NE, BE, SA and LT took part in the conception, study design, investigation, acquisition of data and in supervision, or critically reviewing the article. LT was responsible for the overall publication.

## Funding

The study was funded by Chiesi Hungary Ltd.

## Ethics approval

Local study authorization was provided by the National Institution of Pharmacy and Nutrition of Hungary, based on the beneficial assessment by the National Scientific and Research Ethics Committee of Hungary (docket number: IV/8716–3 /2021/EKU).

## Informed consent statement

As no individual data of patients were recorded during the study, there was no need to gain informed consent forms.

## CRediT authorship contribution statement

**B. Sánta:** Project administration, Methodology, Investigation, Funding acquisition, Formal analysis, Conceptualization. **A. Keglevich:** Supervision, Methodology, Investigation. **T. Kovács:** Supervision, Methodology, Investigation. **B. Engi:** Methodology, Investigation. **A. Südi:** Methodology, Investigation. **E. Noémi:** Supervision, Methodology, Investigation, Formal analysis, Conceptualization. **L. Tamási:** Supervision, Methodology, Investigation, Conceptualization.

## Declaration of competing interest

The authors declare the following financial interests/personal relationships which may be considered as potential competing interests: L. Tamási has received lecture or consultancy fees and/or support for conference attendance from Berlin-Chemie, Orion Corporation, Novartis, Chiesi, Teva Pharmaceutical, and AstraZeneca. N. Eszes has received lecture or consultancy fees and/or support for conference attendance from Berlin-Chemie, Orion Corporation, Novartis, Chiesi, Teva Pharmaceutical, and AstraZeneca.

B. Santa, A. Keglevich and T. Kovács are all employees of Chiesi Hungary Ltd.

A. Südi and B. Engi have nothing to declare.
